# An essential role of the basal body protein SAS-6 in *Plasmodium* male gamete development and malaria transmission

**DOI:** 10.1111/cmi.12355

**Published:** 2014-09-24

**Authors:** Sara R Marques, Chandra Ramakrishnan, Raffaella Carzaniga, Andrew M Blagborough, Michael J Delves, Arthur M Talman, Robert E Sinden

**Affiliations:** 1 Department of Life Sciences, Imperial College of LondonLondon, SW7 2AZ, UK; 2 Institut für Parasitologie, Universität Zürich8057, Zürich, Switzerland; 3 Electron Microscopy Unit, Cancer Research UKLondon, WC2A 3PX, UK; 4 Department of Microbial Pathogenesis, Yale School of Medicine, Boyer Center for Molecular MedicineNew Haven, CT, 06519, USA

## Abstract

Gametocytes are the sole *Plasmodium* parasite stages that infect mosquitoes; therefore development of functional gametes is required for malaria transmission. Flagellum assembly of the *Plasmodium* male gamete differs from that of most other eukaryotes in that it is intracytoplasmic but retains a key conserved feature: axonemes assemble from basal bodies. The centriole/basal body protein SAS-6 normally regulates assembly and duplication of these organelles and its depletion causes severe flagellar/ciliary abnormalities in a diverse array of eukaryotes. Since basal body and flagellum assembly are intimately coupled to male gamete development in *Plasmodium*, we hypothesized that SAS-6 disruption may cause gametogenesis defects and perturb transmission. We show that *Plasmodium berghei* *sas6* knockouts display severely abnormal male gametogenesis presenting reduced basal body numbers, axonemal assembly defects and abnormal nuclear allocation. The defects in gametogenesis reduce fertilization and render *Pbsas6* knockouts less infectious to mosquitoes. Additionally, we show that lack of *Pbsas6* blocks transmission from mosquito to vertebrate host, revealing an additional yet undefined role in ookinete to sporulating oocysts transition. These findings underscore the vulnerability of the basal body/SAS-6 to malaria transmission blocking interventions.

## Introduction

*Plasmodium* is the causative agent of malaria, a deadly disease spread by mosquito vectors. Gametocytes are the only parasite stages transmitted from the host to the mosquito, where sexual reproduction occurs. Briefly, when a mosquito bites an infected host, ingested male and female gametocytes are activated to undergo gametogenesis forming dimorphic motile male microgametes and sessile female macrogametes, which then fertilize forming ookinetes. Ookinetes escape the hostile midgut environment lodging outside the midgut epithelium under the basal lamina, where they will develop into oocysts. Oocysts undergo endomitosis and upon maturation release several thousand sporozoites which invade the salivary glands and can be then injected with the mosquito saliva into the skin of naive hosts, perpetuating the life cycle.

While facultative for the majority of parasite pathogens, gametogenesis and fertilization are obligate steps of the *Plasmodium* life cycle (Heitman, 2010), consequently, disrupting either process prevents infection of new hosts. Because the focus of malaria studies has been to cure the symptoms of disease which are caused by asexual parasites (Fidock, 2010), the molecular aspects of gametogenesis, which solely cause transmission, remain comparatively poorly understood (Guttery *et al*., 2012).

Female gametes differ little from the parental female gametocytes, with gamete development primarily entailing egress from the red blood cell, de-repression and initiation of translation of accumulated mRNAs, as well as expression of surface antigens such as P25 and P28 (Kumar and Carter, 1985; Mair *et al*., 2006). Male gametes differ enormously from parental male gametocytes. Each male gametocyte forms 8 male gametes, which are simple flagellate cells. For successful gamete formation, nucleus and cytoplasm of parental gametocytes have to be exquisitely co-ordinated. In the nuclear compartment, 3 endomitotic divisions produce 8 newly replicated genomes (Sinden *et al*., 1976; Janse *et al*., 1986a,b[Bibr b21],[Bibr b22]). Simultaneously, in the cytoplasmic compartment, an amorphous microtubule organizing centre develops into two planar tetrads of basal bodies (BB), which separate into 8 individual BBs. Each BB serves as a template for one axoneme and remains connected with the genome trough a nuclear pore (Sinden *et al*., 1976; 1978[Bibr b40],[Bibr b41]; Sinden, 1978). The pairing of a single haploid genome/nucleus with each flagellum is critical for the formation of fully functional male gametes. In a process termed exflagellation, the newly assembled individual haploid flagellate gametes are released, BB first, from the residual gametocyte body.

BBs are established platforms for eukaryotic flagella/cilia assembly (Marshall, 2008); considering *Plasmodium* male gametogenesis is so tightly coupled to BB and flagellum assembly, we hypothesized that disrupting the BB would render the parasites infertile and block transmission of the parasite.

To date, there is no published molecular marker for the *Plasmodium* BB and *Plasmodium* genomes contain few conserved BB gene orthologues (Hodges *et al*., 2010; Sinden *et al*., 2010). One of the orthologues encodes SAS-6, which belongs to an ancestral conserved module of proteins that correlates with presence of centrioles/BBs (Carvalho-Santos *et al*., 2010). SAS-6 family members are required for the earliest steps of centriole formation in a range of organisms – from *Chlamydomonas reinhardtii* to *Homo sapiens –* and its depletion often results in failure to form centrioles or produces other centriole abnormalities leading to severe flagellar/ciliary anomalies (Leidel *et al*., 2005; Bettencourt-Dias and Glover, 2009). These anomalies include flagellar absence, loss of flagellar ninefold symmetry and cilia length reduction (Nakazawa *et al*., 2007; Rodrigues-Martins *et al*., 2007; Vladar and Stearns, 2007; Culver *et al*., 2009). SAS-6 has been recently localized to the centriole of *Toxoplasma gondii* suggesting it has a conserved role in Apicomplexa (de Leon *et al*., 2013).

We show that SAS-6 depletion in *Plasmodium berghei* results in reduced BB numbers and abnormal flagellum assembly causing a dramatic reduction in male gametogenesis and fertilization *in vitro*. This is also observed *in vivo*, as mice infected with *Pbsas6* knockout parasites are less infectious to mosquitoes. Surprisingly, we also discovered that knockout parasites do not transmit from mosquitoes to vertebrate host, uncovering an unexpected role for *Pbsas6* in the formation of sporulating oocysts.

## Results

### Key structural motifs of SAS-6 are conserved in *Plasmodium*

**Fig 1:**
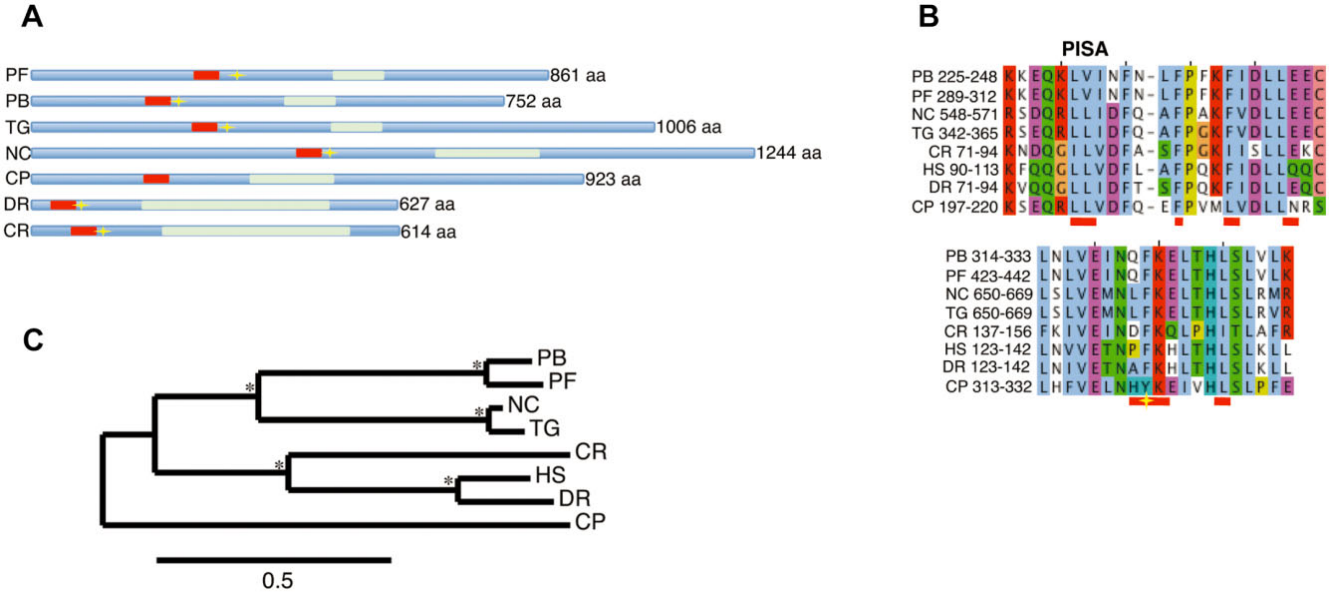
Comparison of malarial SAS-6 with other Apicomplexa and eukaryotes.A. Comparative domain organization of predicted SAS-6 orthologues in apicomplexan and other eukaryote genomes. Red box represents the PISA motif, light green box represents predicted coil-coiled domains, yellow star represents the phenylalanine residue demonstrated to be essential for dimerization in *Danio rerio* (van Breugel *et al*., 2011).B. Multiple sequence alignment of the putative N-terminal oligomerization domain of SAS-6 orthologues. Horizontal red bars indicate the possible interface residues in the N-terminus dimer previously identified in *C. reinhardtii* SAS-6 (Kitagawa *et al*., 2011) Yellow star represents the dimerization residue in *D. rerio* SAS-6 (van Breugel *et al*., 2011).C. Phylogenetic tree of SAS-6 orthologues. Asterisk * signifies bootstrap support greater than 85%. Scale bar stands for number of substitutions per site. PF, *Plasmodium falciparum*; PB, *Plasmodium berghei*; TG, *Toxoplasma gondii*; NC, *Neospora caninum*; CP, *Cryptosporidium parvum*; DR, *Danio rerio*; CR, *Chlamydomonas reinhardtii*; HS, *Homo sapiens*.

The recently published structures of SAS-6 (van Breugel *et al*., 2011; Kitagawa *et al*., 2011) allowed us to make observations on the sequence and structural conservation of malarial SAS-6. These studies show that SAS-6 self-oligomerization can form a ninefold symmetric central-cartwheel structure. Oligomerization occurs via N-terminal association of dimers, at specific residues within the previously defined PISA region (Leidel *et al*., 2005), at an adjacent conserved region as well as within the coil-coiled region of these proteins. We have mapped these regions in *P. berghei* and *P. falciparum* predicted SAS-6 protein and found that the key phenylalanine residue (131 in *Danio rerio*) and a residue comprising the hydrophobic cavity in the PISA region are conserved (Fig. 1A and B). Moreover *Pbsas6* and *Pfsas6* also have a predicted coiled coil domain suggesting that homodimerization can also occur in this region of the protein. Notably, apicomplexan SAS-6 proteins contain additional N and C terminal extensions with no homology to known proteins. Within the apicomplexan phylum many parasite species have lost the ability to form flagella and we were unable to find SAS-6 orthologues in such species (e.g. *Babesia bovis*, *Theileria annulata*). A notable exception is *Cryptosporidium parvum* in which we could find a SAS-6 orthologue (Fig. 1C). However our sequence and phylogenetic analysis revealed that this protein was divergent when compared to other apicomplexan orthologues and that the critical residues were not conserved (Fig. 1A and B). Interestingly *Cryptosporidium* species do not form flagella but do exhibit a BB like structure in their immotile male gametes that seems to nucleate a tubular structure comprised of 9–11 microtubule singlets surrounding the nucleus of the immotile gamete (Ostrovska and Paperna, 1990, Cheadle *et al*., 1999). For coccidian species, which display a conserved classical flagellar structure, SAS-6 was found to have the critical residues (Fig. 1A and B). The observed conservation of residues in apicomplexan organisms that display flagella suggests that SAS-6 homodimerization and function are potentially conserved in the flagellate microgametes of the species of this phylum, with *Pbsas6* and *Pfsas6* likely sharing homologous functions.

### sas6-myc is detected in male gametocytes and its distribution is consistent with a basal body location

**Fig 2:**
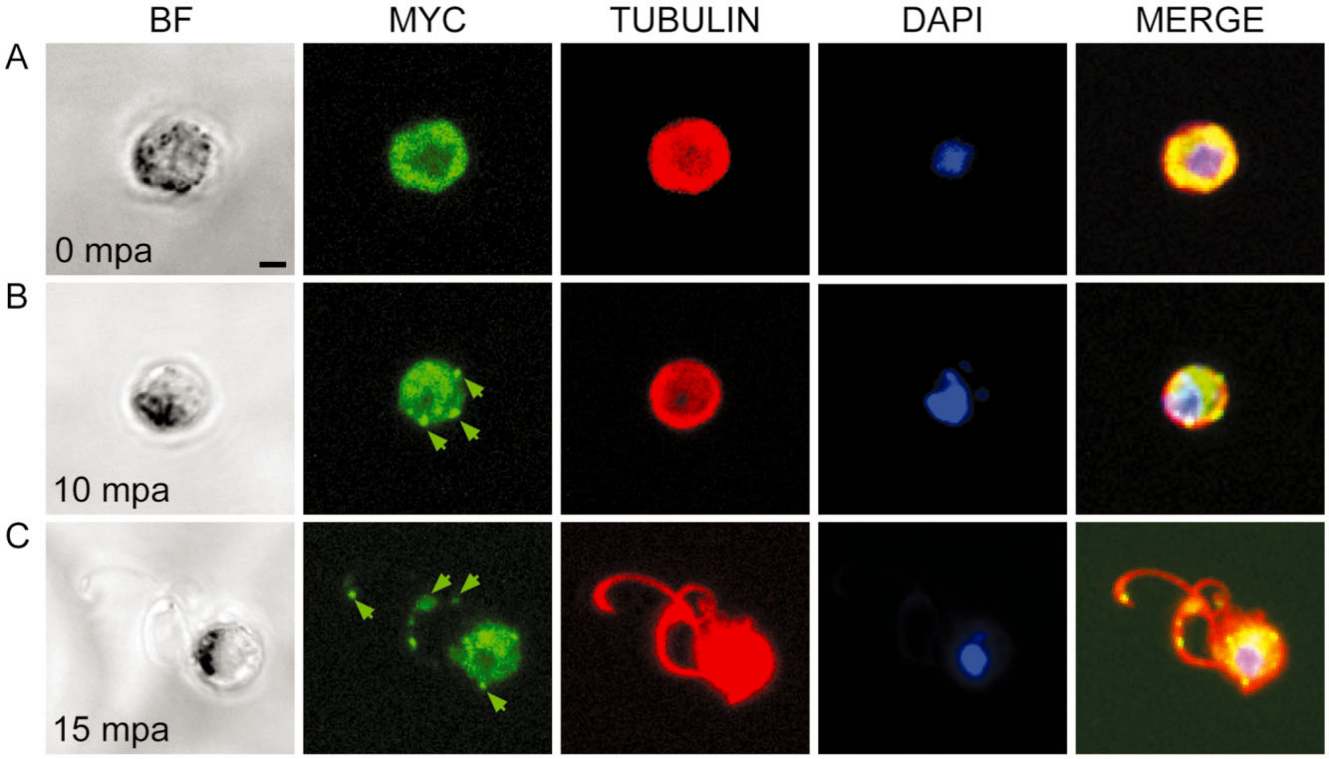
Distribution and location of sas6-myc prior to and during gametogenesis. (A–C). Bright-field and confocal stacks of transgenic sas6-myc gametocytes fixed at different times before and after activation. Immunofluorescence images show DAPI staining of DNA in blue, anti-MYC in green and anti-tubulin in red.A. Before activation, sas6-myc and tubulin are distributed ubiquitously in the cytoplasm of male but not female gametocytes (Fig. S1D).B. At 10 min post-activation (mpa), sas6-myc aggregates in small punctae (green arrow) in the cytoplasm.C. At 15 mpa, sas6-myc is detected in the gametocyte body and at the distal part of the male gametes (green arrows). Scale bars – 2 μm.

**Fig 3:**
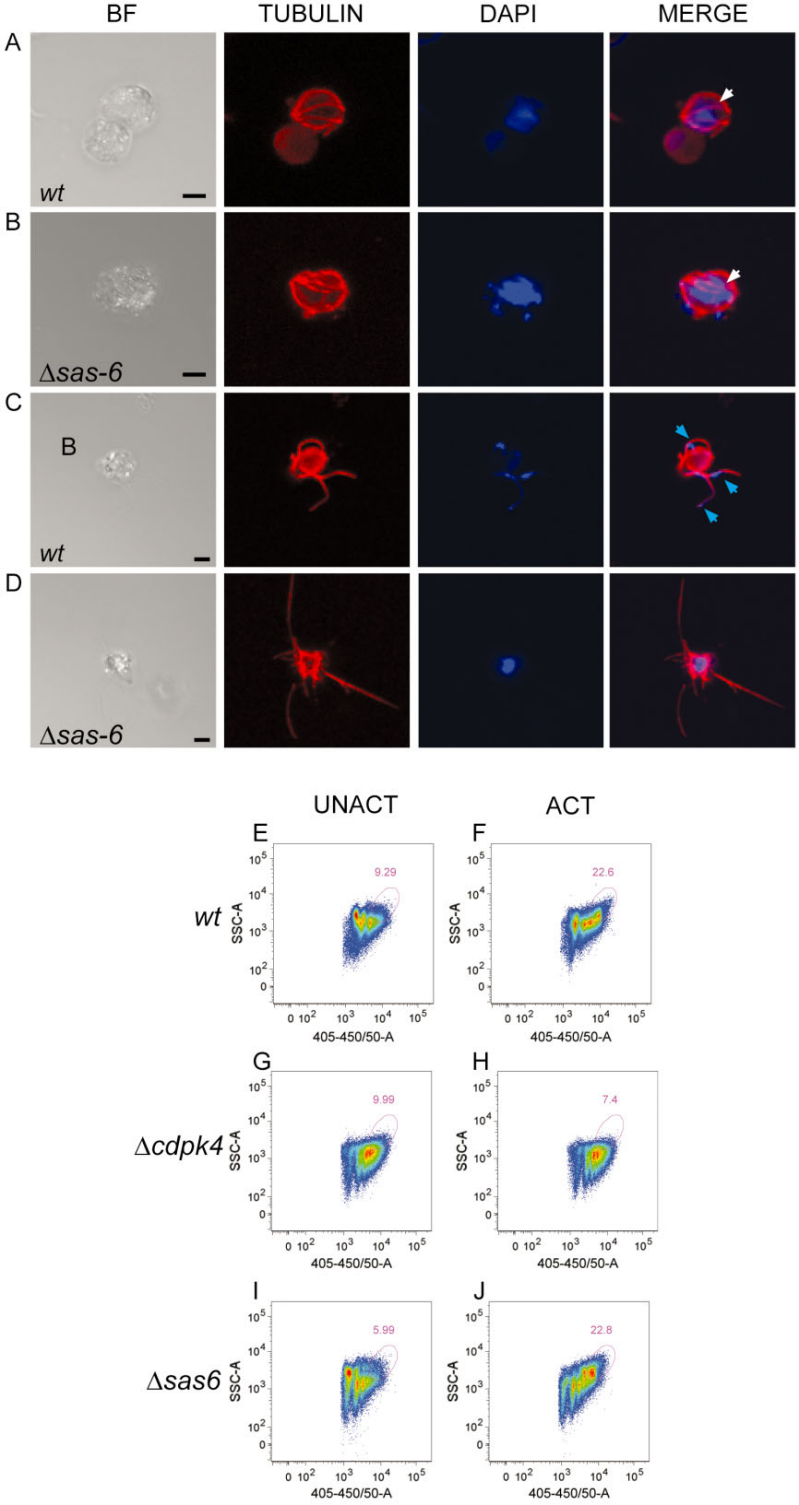
Gametogenesis analysis of Δ*sas6* and wt clones.A–D. Bright field and immunofluorescence images from confocal stacks projections of fixed male gametocytes at 10 and 15 min post activation (mpa). DAPI staining of DNA is in blue and anti-tubulin in red. At 10 mpa wt (A) and Δsas6 (B) are indistinguishable from each other, white arrows point out formed tubulin structures. At 15 mpa, wave-like nucleated gametes are seen outside of the gametocyte body in wt (C), as opposed to Δ*sas6* (D) where tubulin protrusions are thin straight and not nucleated. Blue arrows point out nuclei. Scale bars – 2 μm.E–J. Analysis of gametocyte DNA content by flow cytometry. Representative density plots of Hoechst positive cells of Nycodenz purified gametocyte samples. UNACT represents samples fixed before activation and ACT represents samples fixed at 8 mpa. Plots display intensity of DAPI staining – 405–450/50A versus SSC- sidescatter. Pink gate defines cells with highest DNA content. The percentage of cells with high DNA in unactivated wt gametocyte samples (E) is lower than in activated wt gametocyte samples (F) due to male gametocyte replication. Δ*cdpk4* male gametocytes do not undergo DNA replication (Billker *et al*., 2004) therefore unactivated and activated gametocytes display similar DNA intensity patterns (G, H). Δ*sas6* unactivated gametocyte samples (I) also display a lower percentage of cells with high DNA content when compared to activated gametocyte samples (J) indicating that male gametocyte replication has occurred. Presence of gated cells in Δ*cdpk4* and unactivated preparations most likely represents asexual contamination as observed in Giemsa staining of purified samples. Mean percentage of gated cells upon activation is 9.75 ± 3 for Δ*cdpk4*, 23.35 ± 1 for wt and 24.75 ± 3 for Δ*sas6*.

In most eukaryotes, the centriole/BB body migrates to the plasma membrane where it serves as a template for flagellum assembly (Kobayashi and Dynlacht, 2011). In contrast, *Plasmodium* BBs do not migrate to the membrane remaining closely associated with the nucleus, at least until exflagellation, whereupon the BB portion of the flagellum is the first to emerge from the gametocyte body (Sinden *et al*., 1976; 1978[Bibr b40],[Bibr b41]). To date, there is no *Plasmodium* molecular marker specific for the BB. Since SAS-6 locates to centrioles/BBs in a number of species, we thought that coupling it with a fluorescent protein would provide a useful tool to observe BB behaviour *in vivo*. While several attempts at tagging *Pbsas6* – PBANKA_010620 – with green fluorescent protein failed in our hands, we were able to tag it with myc and analysed protein distribution in transgenic parasites (Fig. S1A–C). As predicted by proteomic studies (Khan *et al*., 2005), sas6-myc is detected by immunofluorescence in male gametocytes and not in females or any other blood stage parasite (Fig. 2A, Fig. S1D). Uniform distribution of sas6-myc in male gametocytes changes upon activation: at 10 min post activation (mpa), we observed distinct punctae of fluorescence varying in number between 4 and 10 per cell (Fig. 2B). Absence of detectable protein in asexual stages and aggregation of protein after activation is consistent with previous electron microscopy data which reported lack of visible centrosome structures in asexuals and *de novo* BB formation after male gametocyte activation (Sinden *et al*., 1976; 1978; Sinden, 1978). At 15 mpa, myc is observed in the male gametocyte body and at the distal tips of exflagellating gametes (Fig. 2C). While the maximum number of punctae does not strictly coincide with the expected maximum number of BB (8), the location of sas6-myc at the protruding tips of the male gametes certainly does (Sinden *et al*., 1976; 1978; Sinden, 1978). We attribute extra-punctae to putative abnormal BB segregation caused by the myc tag. This hypothesis is strengthened by the observation that transgenic gametocytes appear morphologically normal up to this point but display exflagellation abnormalities that mimic aspects of the knockout phenotype. For example, transgenic microgametes frequently lack DNA, a feature that is also observed in *Pbsas6* knockouts (Figs 2C and 3D).

Taken together these results indicate that SAS-6 is a male specific protein, whose distribution is re-organized after male gametocyte activation with a probable BB location.

### *Pbsas6* knockout gametocytes do not form motile nucleated male gametes

*Pbsas6* mRNA expression is detected in asexual as well as sexual and the mosquito stages, except for sporozoites (Fig. S2A). To investigate the function of SAS-6, we generated two clonal populations of *P. berghei* knockout parasites (here called Δ*sas6* and Δ*sas6-gfp*) by double homologous recombination, replacing the *Pbsas6* coding sequence with a drug resistant cassette. Knockout parasites were similarly generated in two different genetic backgrounds (Fig. S2B–D). Asexual growth, gametocyte production and gametocyte sex ratios of Δ*sas6* and Δ*sas6-gfp* clones are indistinguishable from wild-type (wt) (Fig. S2E–G).

Gametocyte activation can be achieved in culture, where exflagellation, the process of male gamete release from the parental gametocyte can be observed by microscopy. One can also observe male gamete wave-like locomotion (Wilson *et al*., 2013) and their adherence to surrounding red blood cells forming so-called ‘exflagellation centres’. In wt parasite preparations, motile microgametes are clearly visible from 10 mpa onwards, whereas in Δ*sas6* preparations we did not detect any motile gametes. At 20 mpa, motile exflagellation centres are formed in wt but are not detected in Δ*sas6* preparations (Table 1).

**Table 1:** Exflagellation analysis of Δ*sas6* and wt clones

Parasite	Exflagellating centers/100 male gametocytes	Male gametocyte nuclear size (au)	Male gametocytes with tubulin protrusions	Released microgametes/100 male gametocytes	Flagella containing DNA
wt	56 ± 13	150 ± 21	48 ± 9%	61 ± 10	92 ± 5%
Δ*sas6*	0*	149 ± 24	28 ± 7%*	2 ± 0.6*	3 ± 0.9%*
wt-gfp	68 ± 23	145 ± 18	56 ± 12%	69 ± 18	93 ± 11%
Δ*sas6-gfp*	0*	153 ± 17	30 ± 14%*	4 ± 1*	5 ± 0.5%*

Mean results of 3 different biological replicates. Motile exflagellating centre analysis was performed in slides using bright-field microscopy at 20 mpa. Nuclear size examination was performed using ImageJ on DAPI stained images of gametocytes fixed at 10 mpa as in Fig. 3. Flagellar protrusion, flagellar release and nuclear size analysis were performed with fluorescent microscopy in gametocytes fixed at 15 mpa as in Fig. 3. au – ImageJ arbitrary units.

* Asterisk indicates statistically significant differences in Student's *t*-test with *P*-values lower than 0.05.

Lack of Δ*sas6* microgamete motility could therefore be due to either absent or malformed flagella. To distinguish between these possibilities, we examined flagellum formation with an anti α-tubulin antibody and simultaneously investigated nuclear organization by DAPI staining. At 10 mpa, wt and Δ*sas6* male gametocytes are indistinguishable from each other: tubulin containing microtubule structures are visible in the cytoplasm and DAPI measurements suggest that DNA replication is normal (Fig. 3A and B; and Table 1). At 15 mpa, wt parasites undergo exflagellation and tubulin stained wt microgametes can be seen either in the process of release from the gametocyte body or already detached from it. Wt flagella usually exhibit wave-like shapes reflecting motility of male gametes (Fig. 3C). Δ*sas6* form tubulin-containing structures that protrude from gametocyte bodies but rarely detach from them (Fig. 3D, Table 1). These tubulin structures, which do not appear to move, display abnormal and linear morphology. We quantified the ratio of male gametocytes with protruding tubulin structures with or without abnormal morphology at 15 mpa and find that Δ*sas6* produces significantly fewer gametocytes with protruding microtubules (28 ± 7%) in comparison with wt (48 ± 9%) (Table 1). Exflagellating wt microgametes usually possess a nucleus and DNA can be easily visualized with DAPI (Fig. 3C, Table 1). In contrast, association of DNA with Δ*sas6* tubulin protrusions is deficient (Fig. 3D, Table 1). Malformed microgametes projecting out of Δ*sas6* male gametocytes rarely contain DNA (3%), as opposed to wt, in which DNA is associated with most flagella detaching or detached from the gametocyte body (92%) (Table 1). Quantification of these parameters was also performed for Δ*sas6-gfp* revealing that both clones share a similar phenotype. To confirm occurrence of DNA replication we analysed the DNA content of purified wt and Δ*sas6* gametocytes by flow cytometry. The DNA profile of wt gametocytes changes upon activation. The population of cells with higher DNA content increases at 8 mpa reflecting male gametocyte DNA replication (Fig. 3E and F). This high DNA content population is not present in Δ*cdpk4* activated gametocytes when compared with unactivated ones because Δ*cdpk4* male gametocytes do not replicate their DNA (Billker *et al*., 2004; Fig. 3G and H). Like wt, Δ*sas6* gametocytes also display a high DNA content population that increases upon activation (Fig. 3I and J).

Taken together these results show that DNA replication occurs and microtubule containing protrusions do form in Δ*sas6* male gametocytes. However, these microgametes are apparently immotile and the vast majority does not contain DNA or most likely a nucleus; therefore we anticipate the majority will be infertile.

### The canonical ‘9 + 2’ microtubule structure of flagella is severely disrupted and basal bodies are rare in Δ*sas6*

Abnormal shape and lack of motility of Δ*sas6* tubulin microgametes suggested that the underlying microtubular structure of axonemes is defective in Δ*sas6* male gametocytes similar to what has been reported in SAS-6 *Chlamydomonas* mutants and *Drosophila* SAS-6 mutant spermatids (Nakazawa *et al*., 2007; Rodrigues-Martins *et al*., 2007).

Flagellum assembly in *Plasmodium* is unusual because it occurs in the cytoplasm and assembly is not dependent upon intraflagellar transport, a rare feature also reported for *Drosophila* sperm (Han *et al*., 2003; Sarpal *et al*., 2003; Briggs *et al*., 2004). Despite divergent assembly, *Plasmodium* flagella retain the canonical microtubule configuration of axonemes: a central microtubule pair surrounded by a rosette of outer doublets, commonly known as a ‘9 + 2’ structure (Sinden *et al*., 1976; 1978[Bibr b40],[Bibr b41]). To further examine the impact of *Pbsas6* depletion in *Plasmodium* we examined the process of male gametogenesis by transmission electron microscopy.

**Fig 4:**
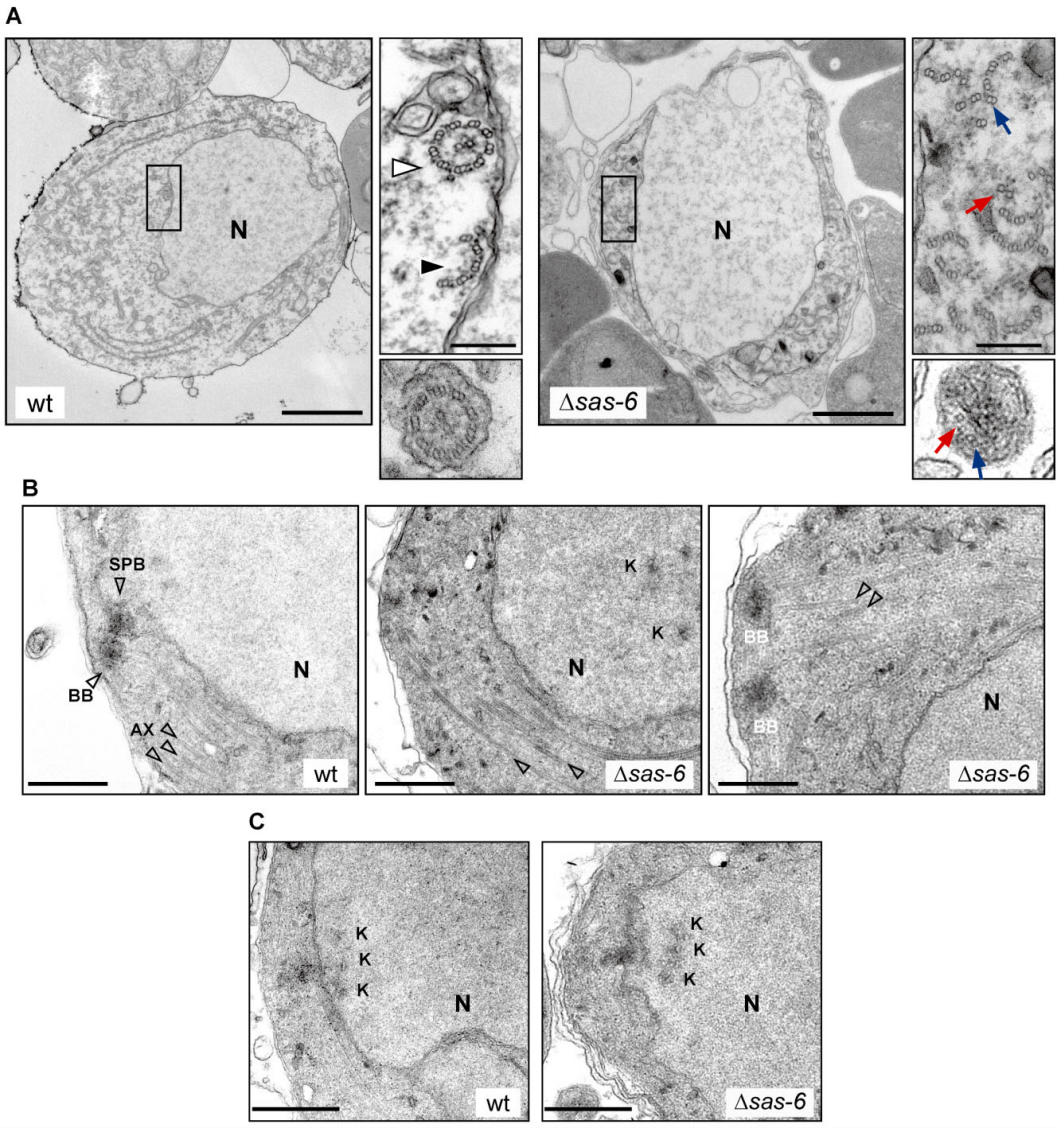
Ultra-structural analysis of gametogenesis of Δ*sas6* and wt clones.A–C. Transmission electron microscopy images of male gametocytes fixed at 15–30 min after activation. (A) Wt and Δ*sas6* activated male gametocytes display an enlarged nucleus (N). The wt inset display a canonical 9 + 2 microtubule rosette (white arrowhead) and an incomplete microtubule rosette (black arrowhead). Δ*sas6* cytoplasmic inset displays a complete lack of canonical ‘9 + 2’ structures with formation of microtubule central pairs (red arrow) and outer microtubule doublets (blue arrow). Cross-section of wt male gamete shows a ‘9 + 2’ structure, cross-section of a Δ*sas6* microtubular protrusion shows lack of normal microtubule patterning. (B) In wt, basal bodies (BB) are present in the cytoplasm and nucleate axonemes (AX) that usually display 3 sets of microtubules (arrowheads), 2 peripheral and 1 central when sectioned longitudinally. BBs connect through a nuclear pore with the spindle-pole-body (SPB). In Δ*sas6*, BBs are rarely seen and longitudinal microtubules show disorganization. Two of the BBs observed in a Δ*sas6* appear normal. (C) Kinetochore (K) appearance in wt and Δsas6 is indistinguishable from each other but in the Δ*sas6*, kinetochores frequently appear detached from the SPB, in a more central position. A scale bars – 2 μm. Inset scale bars – 200 nm. B, C scale bars – 500 nm.

Activated male gametocytes are recognizable by their central rounded nucleus and presence of microtubules in the cytoplasm (Fig. 4A). Wt axonemes display the canonical ‘9 + 2’ rosette structure but rosettes with fewer microtubule doublets are also detected (Fig. 4A inset, Table 2). In contrast, Δ*sas6* microtubule doublets are scattered and complete ‘9 + 2’ rosettes were never observed (Fig. 4A inset, Table 2). Wt microgametes usually display ‘9 + 2’ structure while in Δ*sas6* protrusions display disorganized, abnormal numbers of microtubules (Fig. 4A). These defects are consistent with the observed lack of motility and exflagellation.

**Table 2:** Quantification of microtubular doublet structures, basal bodies, intra-nuclear bodies and kinetochores in Δ*sas6* and wt clones

Genotype	9 + 2	9 + 0	≥ 6 + 2	≥ 6 + 0	4 ≥ db ≤ 6	No. of male cells
wt	116	26	29	19	13	38
Δ*sas6*	0	1	2	6	17	53

Genotype	BB	SPB	KK attached to SPB	KK detached from SPB	No. of male cells
wt	15	8	6	1	120
Δ*sas6*	3	20	8	7	279

Number of microtubule doublets (db) disposed in axoneme like rosettes found in male gametocytes at 30 mpa. Δ*sas6* display lower number of microtubule doublets and 9 + 2 structures were not observed.

Although a direct role for SAS6 in axoneme assembly is possible, the observed defects are consistent with and attributed mainly to the described roles for SAS6 in BB assembly and duplication (Nakazawa *et al*., 2007; Rodrigues-Martins *et al*., 2007). We detected 15 BBs in a total of 120 wt sectioned male cells (12%) (Table 2). In Δ*sas6* however, we mostly observe microtubules doublets that do not extend from recognizable BB structures (Fig. 4B) and out of 279 Δ*sas6* sectioned male cells we only observed 3 BBs (1%) (Table 2). Morphologically, the few Δ*sas6* BBs do not look strikingly different from wt at this resolution (Fig. 4B).

In *Plasmodium*, each BB nucleates one axoneme and connects with the nucleus, namely with a spindle pole body, through a nuclear pore (Fig. 4B). The structural/molecular basis of this connection is unknown but given their close proximity and shared structural components, lack of BBs could impact on spindle function. Activated male gametocytes of wt and Δ*sas6* parasites display similar numbers of spindle poles of indistinguishable morphology (Fig. 4B, Table 2). Kinetochores are very often found close to the nuclear membrane at the spindle pole and their morphology is again indistinguishable between Δ*sas6* and wt cells (Fig. 4C). These results are consistent with the observed lack of detectable DNA replication defects. Interestingly, Δ*sas6* kinetochores were frequently found distant from spindle poles in a more central position in the nucleus (Fig. 4B).

Taken together these results indicate that depletion of SAS-6 results in the formation of fewer BBs, which is most certainly responsible for disruption of canonical axonemal structures and lack of axoneme nucleation. These results indicate that SAS-6 function is conserved in *Plasmodium* and confirm the suspected and crucial role of this protein and BBs in male gametogenesis.

### Abnormal male gametogenesis of Δ*sas6* dramatically decreases fertilization *in vitro*

**Fig 5:**
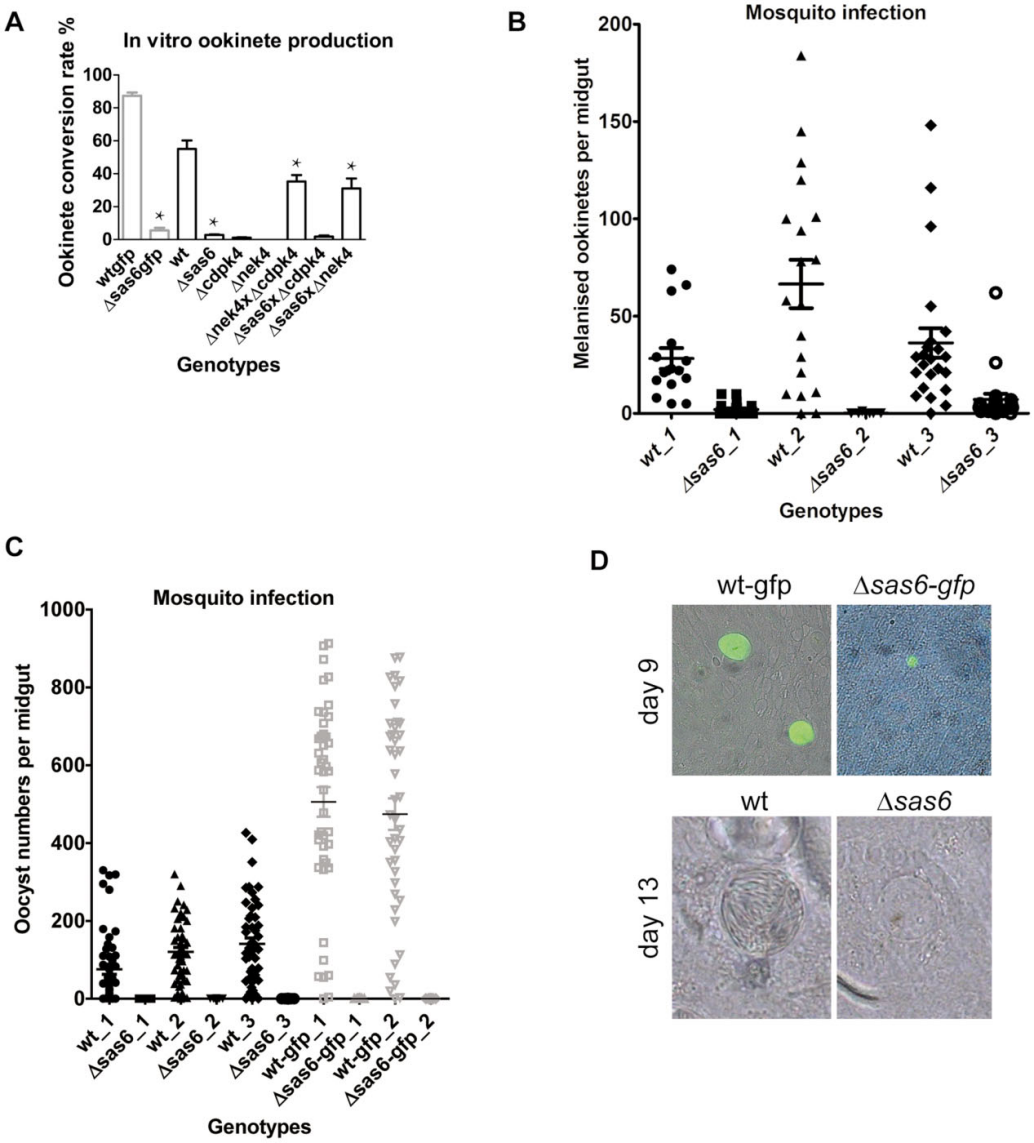
Analysis of sexual reproduction and infectivity to mosquitoes in Δ*sas6* and wt clones.A. Ookinete conversion rate as measured by the number of ookinetes per total number of fertilized and unfertilized females in Δ*sas6-gfp*, Δ*sas6* and crosses of Δ*sas6* with Δ*cdpk4* (male deficient) and Δ*nek4* (female deficient) clones. Δ*sas6* form significantly fewer ookinetes than wt, a phenotype that can be rescued by crossing it with Δnek4. Asterisk * indicates statistically significant differences in Student's *t*-test with *P*-values lower than 0.01, Table S1.B. Ookinete invasion of *An. gambiae* L3-5 midguts. Mosquitoes were infected with wt and Δ*sas6*, mosquitoes were dissected at day 6 and melanized ookinetes were counted in midguts in 3 different biological replicates. Intensity of infection is significantly decreased in all replicates, Table S2A.C. Infectivity of Δ*sas6* to *An. stephensi* mosquitoes. Mosquitoes were infected with wt, wt-gfp, Δ*sas6* and Δ*sas6-gfp*, midguts were dissected at day 12 and oocysts were counted in 3 different biological replicates. Intensity and prevalence of infection are significantly different from wt, Table S2B.D. Bright-field and fluorescent oocyst images at day 9 and day 13. At both time points, Δ*sas6* appear smaller than wt. This size difference was quantified and is statistically significant at day 9 (Table S1). At day 13, wt oocysts display densely packed slender sporozoites that appear like striations, in contrast Δ*sas6* oocysts appear empty.

The location of sas6-myc specifically in male gametocytes suggests that SAS-6 does not have a role in female gametogenesis and we therefore hypothesized Δ*sas6* female gametes should be fertile. Also, while male gametogenesis is severely abnormal, nucleated flagella and 9 + 0 microtubule structures were very occasionally observed (Tables 1 and 2) suggesting self-fertilization might occur. To investigate the impact of *Pbsas6* disruption in gamete fertilization we examined ookinete formation. When ingested in the mosquito blood meal gametocytes rapidly differentiate into gametes, fertilize forming a zygote, which further differentiates into a motile/invasive ookinete. These conditions can be mimicked at very high efficiencies *in vitro* (Yoeli and Upmanis, 1968) and ookinete formation can be quantified as the ratio of activated female gametes expressing P28 that converted into ookinetes. This assay permits genetic crosses between different parasite lines, routinely used to distinguish between male and female specific defects. Here, we crossed Δ*sas6* with either Δ*cdpk4* knockout (Billker *et al*., 2004), which have defective males but normal female gametes, or Δ*nek4* knockout (Reininger *et al*., 2005), which have defective females but normal male gametes. When self-crossed these Δ*cdpk4* and Δ*nek4* clones do not form ookinetes however, crosses between them rescue ookinete production. Considering the severity of the male gametogenesis phenotype it was not surprising to see that self-crosses within Δ*sas6* and Δ*sas6-gfp* clones produce very few ookinetes (Fig. 5A, Table S1). Crosses of Δ*sas6* with Δ*cdpk4* also produce reduced numbers of ookinetes indicating that Δ*cdpk4* fertile macrogametes are not fertilized by Δ*sas6* male gametes. In contrast, crosses of Δ*sas6* with Δ*nek4* rescue ookinete production indicating that Δ*sas6* females are normal can be fertilized by fertile Δ*nek4* male gametes.

### Δ*sas6* infect mosquitoes poorly but do not transmit from mosquitoes to mice

Despite being produced at a very low rate *in vitro*, Δ*sas6* ookinetes appear otherwise normal (Fig. S3A). This suggests that while SAS-6 depletion strongly decreases fertilization, Δ*sas6* might still be infective to the mosquito. To investigate this hypothesis we examined Δ*sas6* ability to infect both mosquitoes and thence mice. We first observed the ability of Δ*sas6* ookinetes to cross the mosquito midgut by using *Anopheles gambiae L3-5*. These are refractory mosquitoes that block ookinete development and subsequently melanize the parasite as it lies under the basal lamina (Collins *et al*., 1986). Melanization renders parasites that cross the midgut easily detectable. *An. gambiae* L3-5 fed on mice infected with wt or Δ*sas6* parasites were dissected 6 days post-feed and melanized ookinetes counted on the midgut. We detected several melanized Δ*sas6* ookinetes indicating these are able to cross the midgut. The intensity of infection (number of ookinetes per midgut) is decreased in Δ*sas6* when compared to wt (Fig. 5B, Table S2A). To test the ability of Δ*sas6* and Δ*sas6-gfp* ookinetes to produce oocysts, we allowed fully susceptible *An. stephensi* mosquitoes to feed on infected mice, dissected their midguts 12 days later and counted the number of parasites per midgut. Wt and wt-gfp parasites formed numerous oocysts which contained sporozoites, mean numbers ranging from 75 to 505 oocysts per midgut (Fig. 5C, Table S2B). In contrast, we detected very few Δ*sas6* and Δ*sas6-gfp* oocysts, averages ranging from 0 to 0.06 per midgut. Both prevalence and intensity of infection are significantly decreased compared to wt (Table S2B). Δ*sas6* oocysts also have significantly smaller diameters than wt at day 9 (Fig. 5D, Table S1). At day 12/13, sporozoites are clearly visible inside wt and wt-gfp oocysts by bight-field microscopy, while Δ*sas6* and Δ*sas6-gfp* oocysts are patently devoid of sporozoites (Fig. 5D). Differences between wt and Δ*sas6* oocysts are further confirmed by examining DNA content: DAPI staining of midgut sections reveals that Δ*sas6* oocysts display diffuse DNA rather than punctated DNA (Fig. S3B). These results suggest that genome replication and division in Δ*sas6* oocysts is defective. Confirmation of the latter was found when we failed to detect sporozoites in salivary glands of mosquitoes infected with Δ*sas6*; therefore Δ*sas6* may be unable to transmit from mosquitoes to mice. To test this hypothesis, *An. stephensi* mosquitoes fed 21 days previously on mice infected with wt or Δ*sas6* knockouts were allowed to bite naïve mice. Ensuing parasitaemia was examined by Giemsa staining of blood daily after bite. 247 mosquitoes fed on either Δ*sas6* and Δ*sas6-gfp* infected mice did not transmit the parasite to any of the 7 bitten naïve mice (Table 3). In contrast, 114 mosquitoes fed on wt and wt-gfp infected mice transmitted the parasite to 6 of 6 naive mice. To rule out the possibility that Δ*sas6* and Δ*sas6-gfp* transmit less efficiently due to low ookinete and thereafter low oocyst and sporozoite production, we produced wt and knockout ookinetes *in vitro* and fed these to mosquitoes at similar concentrations. Δ*sas6* and Δ*sas6-gfp* oocyst prevalence at day 9 is comparable to wt but the prevalence of Δ*sas6* oocysts at day 13 is significantly reduced (Fig. S3C, Table S3). While the artificially lowered numbers of ookinetes reduce wt transmission (only 2 of 3 mice infected), even concentrated Δ*sas6* ookinetes still cannot transmit to mice (0 of 3 mice infected) (Table 4).

**Table 3:** Analysis of transmission of Δ*sas6* by direct feed

Parasite	Mouse identity #/# fed mosquitoes	Mouse parasitaemia
wt	1/31	Positive
	2/29	Positive
	3/25	Positive
Δ*sas6*	1/36	Negative
	2/40	Negative
	3/37	Negative
wt-gfp	1/10	Positive
	2/12	Positive
	3/17	Positive
Δ*sas6-gfp*	1/25	Negative
	2/37	Negative
	3/32	Negative
	4/30	Negative

For each replicate, mice with similar parasitaemias were fed to *An. stephensi* mosquitoes. Fully fed mosquitoes were kept for 21 days and allowed to bite on naïve TO mice. Infection of naïve mice was examined by Giemsa staining of the blood after the bite.

**Table 4:** Analysis of transmission of Δ*sas6-gfp* by ookinete feed

Parasite	Mouse identity #/# fed mosquitoes	Mouse parasitaemia
wt-gfp	1/28	Positive
	2/27	Negative
	3/25	Positive
Δ*sas6-gfp*	1/26	Negative
	2/41	Negative
	3/32	Negative

For each replicate, the same number of ookinetes was put into membrane feeders and *An. stephensi* mosquitoes were allowed to feed. Fully fed mosquitoes were kept for 21 days and allowed to bite on naïve C57BL/6 mice. Infection of naïve mice was examined by Giemsa staining of the blood.

Taken together these results indicate that SAS-6 is required for oocyst development. Knockout oocysts display growth failure, inability to rearrange their DNA and to form sporozoites being therefore unable to transmit to the next mouse host.

## Discussion

### A crucial role for *Pbsas6* during male gametogenesis

**Fig 6:**
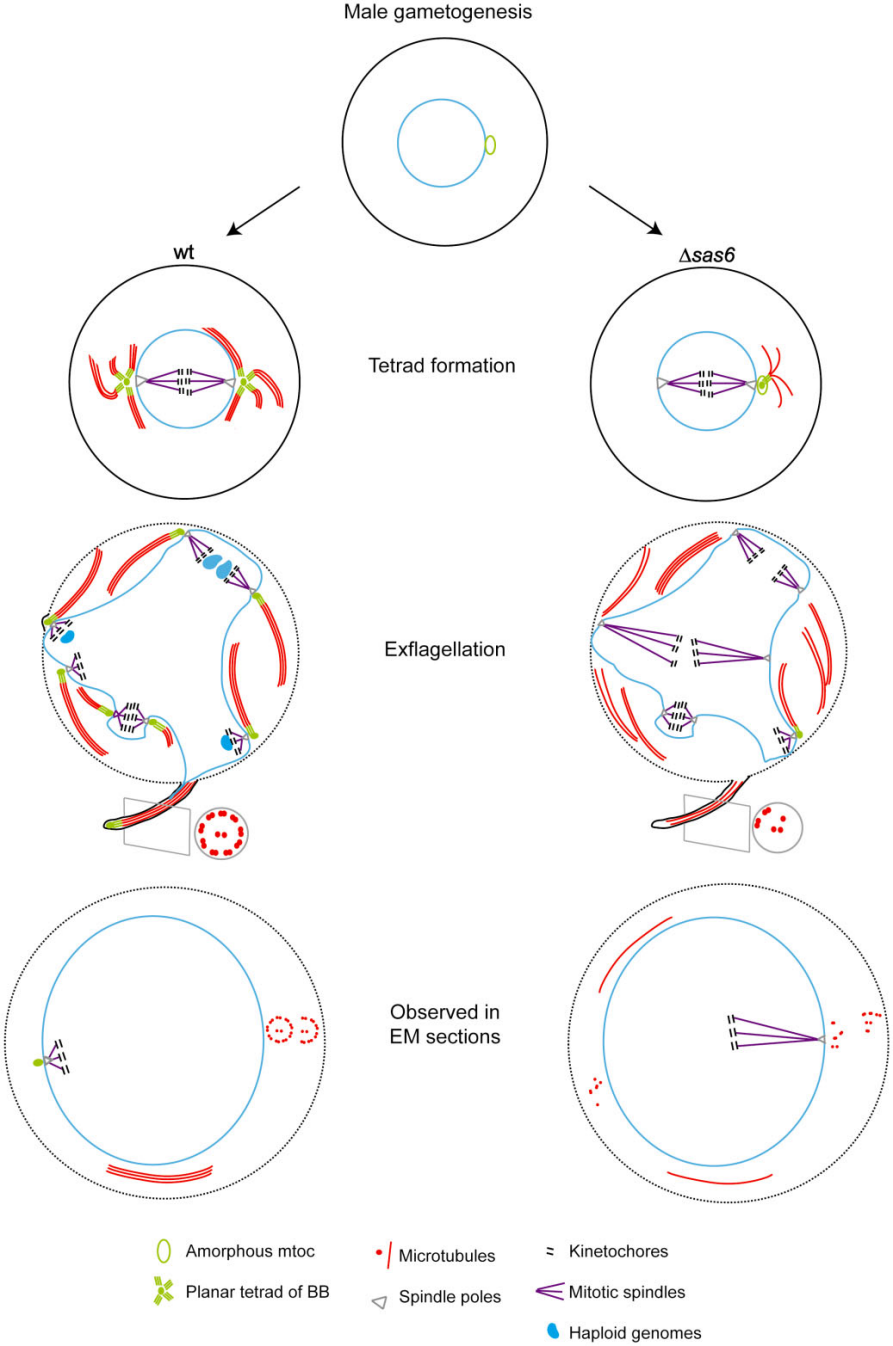
Schematic model of the impact of *Pbsas6* deletion on male gametogenesis.Upon activation, the wt amorphous microtubule organizing centre (mtoc) develops into two planar tetrads of BBs (green), which later separate into 8 individual BBs. Each BB serves as a platform for axonemal microtubule (red) assembly. BB individualization is concomitant with 3 rounds of mitotic division, with BBs connecting with mitotic spindles (purple) and kinetochores (black) via the spindle pole body (grey). Haploid genomes (blue) connect with BBs and are pulled into the emerging wt microgametes, which display a ‘9 + 2’ structure. Δ*sas6* gametocytes display reduced numbers of BBs and lack of ‘9 + 2’ canonical axonemal structures. Axonemal defects are likely due to abnormal formation or segregation of BBs. Abnormal BB production probably disrupts the link between axonemal microtubules and genomes, therefore abnormal Δ*sas6* microgametes rarely contain DNA.

Our results show an essential and conserved role for SAS-6 in *Plasmodium* BB and flagellum assembly. Additionally, we reveal the dramatic impact of its depletion on male gamete development, fertilization, oocyst development and transmission. Immunofluorescence and electron microscopy results combined, allowed us to develop a schematic model for the impact of *Pbsas6* depletion on male gametogenesis (Fig. 6).

In activated wt gametocytes, an amorphous microtubule organizing centre forms two planar tetrads of BBs which are connected with the spindle apparatus via nuclear pores. In the subsequent rounds of mitotic division, the 8 BBs segregate with the newly replicated genomes. During exflagellation, each BB maintains the linkage between one axoneme and one haploid genome forming individual male gametes. Δ*sas6* gametocytes display reduced BB numbers and lack of proper axonemal ‘9 + 2’ assemblies (Fig. 4A). Interestingly, Δ*sas6* do form microtubule doublets and central pairs indicating that the initiation of microtubule assembly *per se* is normal. Since microtubule polymerization precedes separation of the ‘daughter’ BBs (Sinden *et al*., 1976), we speculate that the reduced number of patterned microtubule structures (axonemes) is likely to be due to defective formation of proper BB numbers, abnormal segregation of the BBs or perhaps a combination of both. While a direct role for SAS-6 in flagellum assembly cannot be excluded, it is likely that BB defects cause the observed microtubule/axonemal anomalies.

Low BB numbers do not impact on DNA replication, the number of spindle poles or presence of kinetochores (Tables 1 and 2), therefore we hypothesize that mitotic spindles are normal. Interestingly, the frequent central position of kinetochores (Fig. 4B) is consistent with a prolonged metaphase. In the absence of proper BB numbers, the link between cytoplasmic microtubule structures and genomes is missing, preventing haploid genomes from being included into emerging microgametes (Fig. 3B, Table 1). Abnormal microtubule structures most likely underlie loss of flagellar motility. Abnormal motility coupled to lack of genome inclusion likely causes the observed dramatic reduction of fertilization.

We have also found a small number of microgametes with associated DNA and some 9 + 0 axonemes (Table 1) but it is still difficult to conceive how these immotile gametes can fertilize. One hypothesis is if gametes are by chance physically touching, membrane fusion of gametes of opposite sex mediated by fusogenic proteins like HAP2 (Liu *et al*., 2008) – which is present in Δ*sas6* (Fig. S3D) – can prevail over lack of motility.

Rescue of the fertilization phenotype by crossing Δ*sas6* with Δ*nek4* shows a requirement for SAS-6 in male gametocytes/gametes consistent with its distribution in these cells (Fig. 5A). SAS-6 aggregation after activation and localization at flagellum tips suggest a conserved BB location (Fig. 2) and fits with the structural defects found in the knockouts. Moreover, this distribution is in harmony with previous electron microscopy data suggesting that in *Plasmodium* the BB forms *de novo* after gametocyte activation (Sinden *et al*., 1976; 1978).

Male gametogenesis is particularly attractive target from a therapeutic perspective. Male gametes develop in an evolutionary divergent way (Sinden *et al*., 2010) so putative targets of malaria male gametogenesis are less likely to interfere with host biology. Moreover, male gametogenesis is more sensitive than female gametogenesis to disruption by anti-malarial drugs (Delves *et al*., 2013). This is most likely due to the fundamental, targetable yet poorly understood biology that underlies male gamete development. The crucial role of flagella in the life cycle of many parasite species that cause human disease has been recognized and screening of compounds that prevent SAS-6 oligomerization further validates the prominence of this protein as a therapeutic target (van Breugel *et al*., 2014).

### A crucial role for *Pbsas6* after fertilization

The few Δ*sas6* ookinetes that cross the midgut do not undergo normal sporogony. These oocysts are smaller, display abnormal DNA distribution and fail to form sporozoites, therefore not transmitting from mosquitoes to naïve mice (Tables 3 and 4). The small oocyst size observed suggests an early developmental arrest which might be coupled to or causing defects in DNA replication. Evidence of a cytoplasmic microtubule organizing centre in the developing oocyst was never found (Schrevel *et al*., 1977; Sinden, 1978) but one appealing hypothesis could be that SAS-6 is required during oocyst development for proper chromosome segregation and allocation, in the process of rapid mitosis somewhat similar to that observed during male gametogenesis (Schrevel *et al*., 1977; Janse *et al*., 1986b). The specific mechanism of action, the exact stage at which SAS-6 is required in this transition and whether it is required in the remaining unobserved mosquito and liver stages remains to be determined. The future development of antibodies against *Plasmodium* SAS-6 will allow examination of protein location in mosquito stages as well as determine if the discrepancy between RNA expression and myc detection are due to translational regulation or sensitivity of myc fusion detection. The generation of conditional knockouts will allow depletion of the protein in a temporally controlled manner simultaneously providing larger numbers of knockouts to examine.

Our work not only reveals the crucial and conserved role of *Pbsas6* in flagellum assembly and gametogenesis but also explores its impact on transmission and uncovers a novel role for this gene in oocyst development.

## Experimental procedures

### Immunocytochemistry

Samples were fixed at different time points in fresh 4% paraformaldehyde (PFA). The suspension was allowed to adhere onto poly-l-lysine coated slides overnight at 4°C. The slides were washed once with PBS and immunocytochemistry performed per manufacturer's instructions for rabbit monoclonal anti-myc 1:200 (Cell Signaling). Mouse monoclonal anti-alpha tubulin 1:500 (Sigma) was used simultaneously or independently using the same protocol. Secondary antibodies Alexa 488-conjugated anti-rabbit IgG and Alexa 568 conjugated anti-mouse IgG for fluorescence detection were used at 1:1000 (Molecular probes). The slides were mounted in Vectashield with DAPI (Vector Labs). Parasites were visualized on a Leica SP5 confocal microscope and acquired and analysed with the LAS AF Lite software (Leica). For quantification, samples were visualized on a Leica DMR microscope and imaged with the Zeiss AxioCam HRC and Axiovision software respectively. Cell nuclei diameters were measured using ImageJ and statistical analysis performed using Student's *t*-test.

### Flow cytometry

Gametocytes were Nycodenz purified from infected blood and either immediately fixed (unactivated) or transferred to standard ookinete culture medium for activation and fixed at 8 mpa. Cells were fixed in 4% PFA for 20 min, washed in PBS and stained for 30 min with Hoechst 33342 at a concentration of 0.5 μg μl^−1^. Hoechst-fluorescence intensity was analysed by FACS using a BD LSR Fortessa cytometer. 50 000 cells were analysed per sample on 4 biological replicates. Data analysis was performed using FlowJo.

### Electron microscopy

Gametocytes were purified, fixed at 4°C and processed as previously described (Talman *et al*., 2011).

### Parasite production and purification and mosquito infection

*P. berghei* was maintained by cyclic passage in 6- to 8-week-old female Tucks Ordinary (TO). To induce hyper-reticulocytosis, mice were treated intraperitoneally (i.p.) with 0.2 ml of 6 mg ml^−1^ phenylhydrazine (BDH Chemicals Ltd, UK) 2–3 days prior to parasite i.p. inoculation. Δ*cdpk4* is RMgm-12, Δ*nek4* is RMgm-60. Details on tagging and knockout of PBANKA_010620 production are shown in Figs S1 and 2.

### Parasite crosses and ookinete production

At day 3 post infection of phenylhydrazine treated mice, parasite Δ*sas6*, Δ*sas6-gfp*, Δ*nek4*, Δ*cdpk4* and wt were harvested by heart puncture and mixed at a 1:1 ratio in ookinete medium. After 24 h, ookinete conversion assays were performed as previously described (Tewari *et al*., 2005) by incubating samples with 13.1 antibody (antibody against Pb28 conjugated with Cy3). The proportion of ookinetes to all 13.1-positive cells (unfertilized macrogametes and ookinetes) was established, counting fields at 60 × magnification. Experiments were made in biological triplicate.

### Transmission experiments

For mosquito infections, 3- to 8-day-old female adult *An. stephensi* and *An. gambiae* mosquitoes reared as previously described (Dimopoulos *et al*., 1998) were allowed to feed on anaesthetized infected mice for 20 min at day 3 post infection or fed cultured ookinetes (Rodriguez *et al*., 2002; Sinden *et al*., 2002). Unfed mosquitoes were removed 1 day after feeding. Mosquitoes were dissected for oocysts counts on midguts at day 6 (for refractory mosquitoes) or day 12 post blood feeding. Alternatively mosquitoes were allowed to bite anaesthetized mice at day 21 post feeding. Blood smears from bitten mice were analysed for 14 days following mosquito bite to determine parasitaemia. Experiments were performed in triplicate.

### Bioinformatics and phylogenetic analysis

SAS-6 orthologues were identified in the OrthoMCL database and verified by reciprocal BLAST searches (Altschul *et al*., 1997). A multiple sequence alignment was generated using MUSCLE (Edgar, 2004) and curated with GBLOCKS (Castresana, 2000). Phylogenetic analyses were performed using the curated alignment using PhylM (Dereeper *et al*., 2008). The phylogenetic tree was constructed with the maximum likelihood method under the WAG model.

## Supporting Information

**Fig. S1.** (A) Schematic representation of *Pbsas6* locus before and after insertion of a myc tag by single homologous recombination with the C-terminal part of the gene (PBANKA_010620). The sas6-myc plasmid was generated by amplifying the final 923 bp of the SAS-6 coding sequence with primers E and F, a unique restriction site for *EcoRV* was inserted by nested PCR in the middle of this region to allow for single digestion and single crossover (blue vertical bar). This was inserted into pOB150 (courtesy of O. Billker). The stop codon was removed and the myc coding sequence was attached to it, the plasmid also contained the *T. gondii* dhfr resistance marker. (B) PCR and Southern blot analysis of tagged clone genomic DNA after plasmid integration. Primer positions are represented by black numbered arrows in the locus schematics. Construct integration was confirmed by PCR using primer 9 upstream of the amplified region and primer 10 in the myc construct. Primers 3 and 4 confirm presence of the resistant cassette. Integration was further confirmed by southern blot upon digestion of genomic DNA with *NsiI* (pale green) and *SciI* (dark green) which originates fragments of different sizes for transgenic (1.6 and 4.6 kb) and WT (1.9 kb). The southern probe (horizontal red bar) was amplified with primers 7 and 8. Primer sequences can be seen in Table S3. (C) Western blot analysis of sas6-myc. Under reducing conditions, an anti-myc antibody recognizes a protein of approximately 90 kDa size consistent with the predicted molecular weight of the tagged protein. Mixed asexuals and sexual stages – mix, purified unactivated gametocytes – UG and purified activated gametocytes – AG. (D) sas6-myc distribution in blood stages. Bright-field and fluorescence images of a typical staining, anti-myc in green can only be detected in male gametocytes. Male – M, Female – F, Asexual – AS.

**Fig. S2.** (A) Expression analysis of *Pbsas6* in asexuals, activated gametocytes, ookinetes, oocysts and sporozoite stages by RT-PCR on an intronic region. Genomic DNA was used to show differences between genomic DNA and RNA transcript. Tubulin was used as a loading control. Primers sequences in Table S3. (B) Schematic representation of *Pbsas6* locus before and after removal of the coding sequence by inserting *Toxoplasma gondii* dihydrofolate reductase thymidilate synthase (*Tgdhfr-ts*) gene through homologous recombination with the 3′ UTR and 5′ UTR of *Pbsas6*. The KO construct contained *Pbsas6* 5′ and 3′ UTRs flanking Tgdhfr, which confers resistance to pyrimethamine. The KO plasmid pΔsas6 was generated by amplification of 717 bp of the 5′ UTR (Primers A and B) and 860 bp of the 3′ UTR (primers C and D) of the Pbsas6 coding sequence (PBANKA_010620). These fragments were inserted into pOB90 (courtesy of O. Bilker) on either side of the resistance gene. The fragment was electroporated into *P. berghei* wt ANKA 2.34 and *P. berghei* wt-gfp 507cl1-GFP as previously described. Following drug selection independent clonal populations of each genetic background were selected by limiting dilution. (C) Representative analysis of knockout clone genomic DNA after plasmid integration by PCR. Primer positions are represented by black numbered arrows in the locus schematics. PCRs to test integration of the construct in the correct locus used primer 1 upstream of the genomic sequence and primer 2 inside the construct, as well as primer 5 inside the construct and primer 6 downstream of the genomic sequence. Amplification with primers 3 and 4 confirms the presence of the resistance cassette, amplification with primers 7 and 8 confirm absence of coding sequence in the knockout. (D) Analysis of knockout clone genomic DNA after plasmid integration by Southern blot. Restriction of genomic DNA with *SwaI* (red arrow) and *HincII* (orange arrow) produces fragments of different sizes for knockout (1.4 kb) and wild-type (2.2 kb) DNA. The southern probe (horizontal orange bar) was made by amplification of the 5′ UTR region (primers A and B). (E) Asexual and sexual growth of Δ*sas6* and wt in Giemsa stained blood smears. For asexuals, 3 mice per genotype were infected with 1000 parasites each and parasitemias were examined every day post infection. At day 8 post infection, parasitemias are indistinguishable between wt and Δ*sas6*. Number and sex of gametocytes was determined on infections of 3 mice per genotype treated with phenylhydrazine and infected with similar parasite number per genotype. At day 4, gameotcytaemias and sex ratio are indistinguishable between wt and Δ*sas6*.

**Fig. S3.** (A) Giemsa and P28 stainings using 13.1 labelled Cy3 of activated females and ookinetes at 24 h post fertilization (hpf). Δ*sas6* activated females and ookinetes display regular morphology and express P28 suggesting these parasite stages are indistinguishable from wt. (B) Oocyst images at day 12/13. Infected midguts were embedded in agar and wax, 4 micron sections were taken dehydrated and stained with DAPI. Wt oocysts display wide diameters and their DNA displays a distinct punctated arrangement, Δsas6 oocysts are smaller and DNA appears disorganized and diffuse. Dotted lines outline the oocysts (C) Ookinete fed mosquito infections, oocyst counts were performed at day 9 and 13 post-feed, prevalence and ookinete concentration is shown in Table S3 (D) Hap2 presence in purified activated gametocytes. Westernblot analysis showing that fusogenic protein Hap2 is present in Δ*sas6*.

**Table S1.** *In vitro* ookinete conversion assay. **Table S2A.** Intensity and prevalence of mosquito infections – day 6. **Table S2B.** Intensity and prevalence of mosquito infections – day 12. **Table S3.** Intensity and prevalence of mosquito infections from ookinete feeds. **Table S4.** Cloning, diagnostic integration and RT primers.
