# Supplementary figures and images for: An essential role of the basal body protein SAS-6 in Plasmodium male gamete development and malaria transmission
Source: Cell Microbiol. 2014 Sep 24;17(2):191–206. doi: 10.1111/cmi.12355 (PMC4441282; doi:10.1111/cmi.12355)

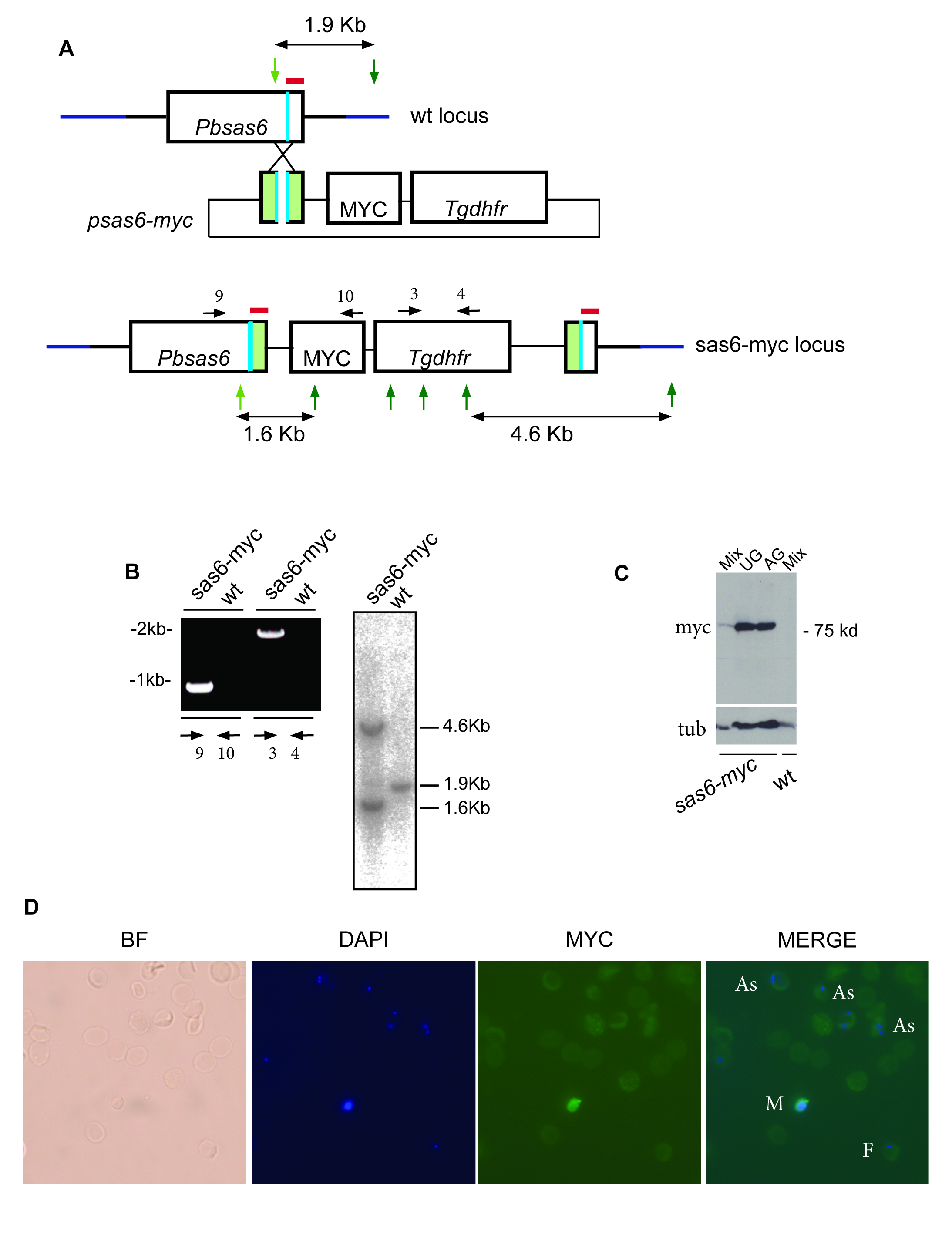

Supplement: Supplementary file 1 — Fig. S1. (A) Schematic representation of Pbsas6 locus before and after insertion of a myc tag by single homologous recombination with the C-terminal part of the gene (PBANKA_010620). The sas6-myc plasmid was generated by amplifying the final 923 bp of the SAS-6 coding sequence with primers E and F, a unique restriction site for EcoRV was inserted by nested PCR in the middle of this region to allow for single digestion and single crossover (blue vertical bar). This was inserted into pOB150 (courtesy of O. Billker). The stop codon was removed and the myc coding sequence was attached to it, the plasmid also contained the T. gondii dhfr resistance marker. (B) PCR and Southern blot analysis of tagged clone genomic DNA after plasmid integration. Primer positions are represented by black numbered arrows in the locus schematics. Construct integration was confirmed by PCR using primer 9 upstream of the amplified region and primer 10 in the myc construct. Primers 3 and 4 confirm presence of the resistant cassette. Integration was further confirmed by southern blot upon digestion of genomic DNA with NsiI (pale green) and SciI (dark green) which originates fragments of different sizes for transgenic (1.6 and 4.6 kb) and WT (1.9 kb). The southern probe (horizontal red bar) was amplified with primers 7 and 8. Primer sequences can be seen in Table S3. (C) Western blot analysis of sas6-myc. Under reducing conditions, an anti-myc antibody recognizes a protein of approximately 90 kDa size consistent with the predicted molecular weight of the tagged protein. Mixed asexuals and sexual stages – mix, purified unactivated gametocytes – UG and purified activated gametocytes – AG. (D) sas6-myc distribution in blood stages. Bright-field and fluorescence images of a typical staining, anti-myc in green can only be detected in male gametocytes. Male – M, Female – F, Asexual – AS. [file cmi0017-0191-sd1.tif]

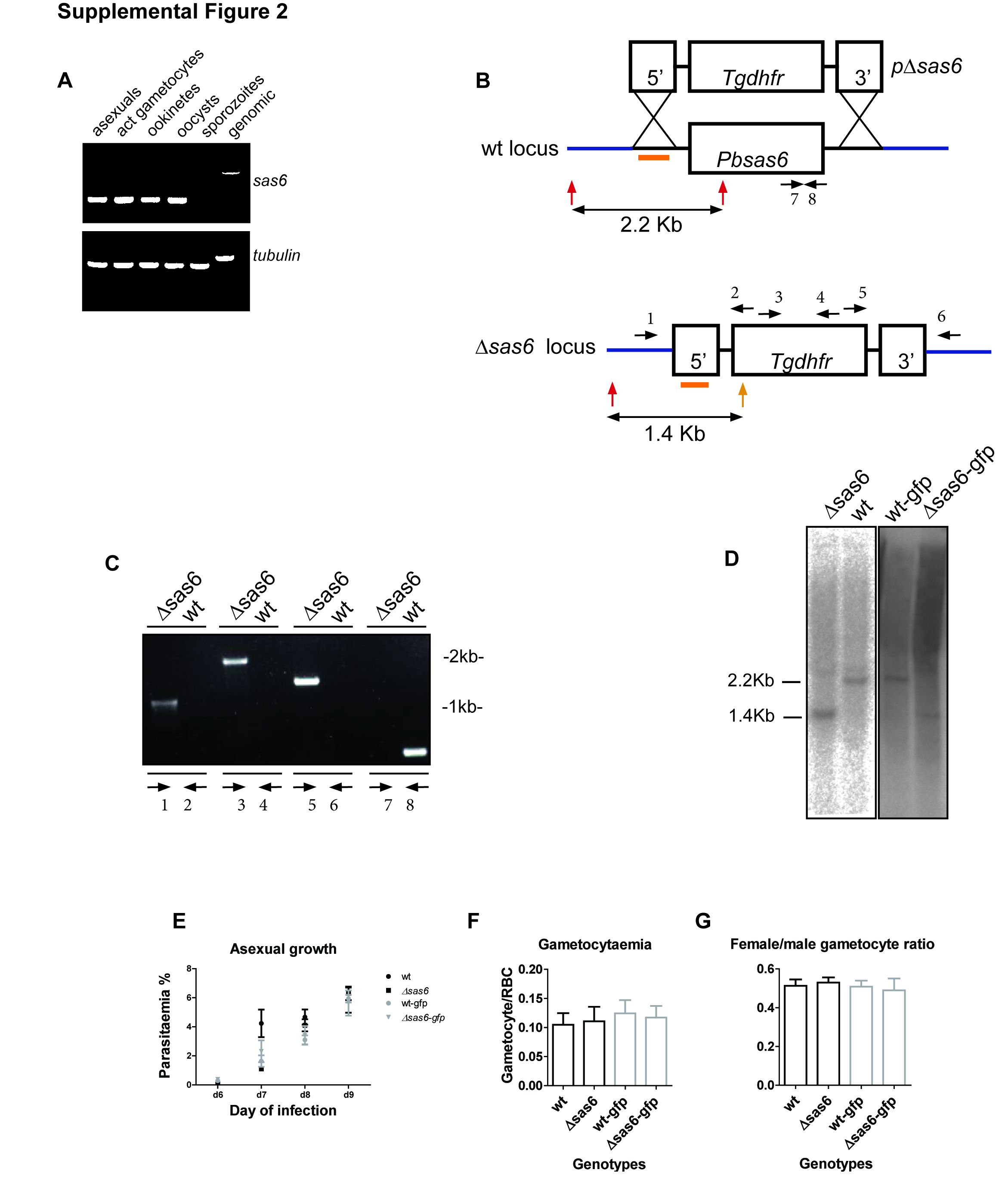

Supplement: Supplementary file 2 — Fig. S2. (A) Expression analysis of Pbsas6 in asexuals, activated gametocytes, ookinetes, oocysts and sporozoite stages by RT-PCR on an intronic region. Genomic DNA was used to show differences between genomic DNA and RNA transcript. Tubulin was used as a loading control. Primers sequences in Table S3. (B) Schematic representation of Pbsas6 locus before and after removal of the coding sequence by inserting Toxoplasma gondii dihydrofolate reductase thymidilate synthase (Tgdhfr-ts) gene through homologous recombination with the 3′ UTR and 5′ UTR of Pbsas6. The KO construct contained Pbsas6 5′ and 3′ UTRs flanking Tgdhfr, which confers resistance to pyrimethamine. The KO plasmid pΔsas6 was generated by amplification of 717 bp of the 5′ UTR (Primers A and B) and 860 bp of the 3′ UTR (primers C and D) of the Pbsas6 coding sequence (PBANKA_010620). These fragments were inserted into pOB90 (courtesy of O. Bilker) on either side of the resistance gene. The fragment was electroporated into P. berghei wt ANKA 2.34 and P. berghei wt-gfp 507cl1-GFP as previously described. Following drug selection independent clonal populations of each genetic background were selected by limiting dilution. (C) Representative analysis of knockout clone genomic DNA after plasmid integration by PCR. Primer positions are represented by black numbered arrows in the locus schematics. PCRs to test integration of the construct in the correct locus used primer 1 upstream of the genomic sequence and primer 2 inside the construct, as well as primer 5 inside the construct and primer 6 downstream of the genomic sequence. Amplification with primers 3 and 4 confirms the presence of the resistance cassette, amplification with primers 7 and 8 confirm absence of coding sequence in the knockout. (D) Analysis of knockout clone genomic DNA after plasmid integration by Southern blot. Restriction of genomic DNA with SwaI (red arrow) and HincII (orange arrow) produces fragments of different sizes for knockout (1.4 kb) [file cmi0017-0191-sd2.tif]

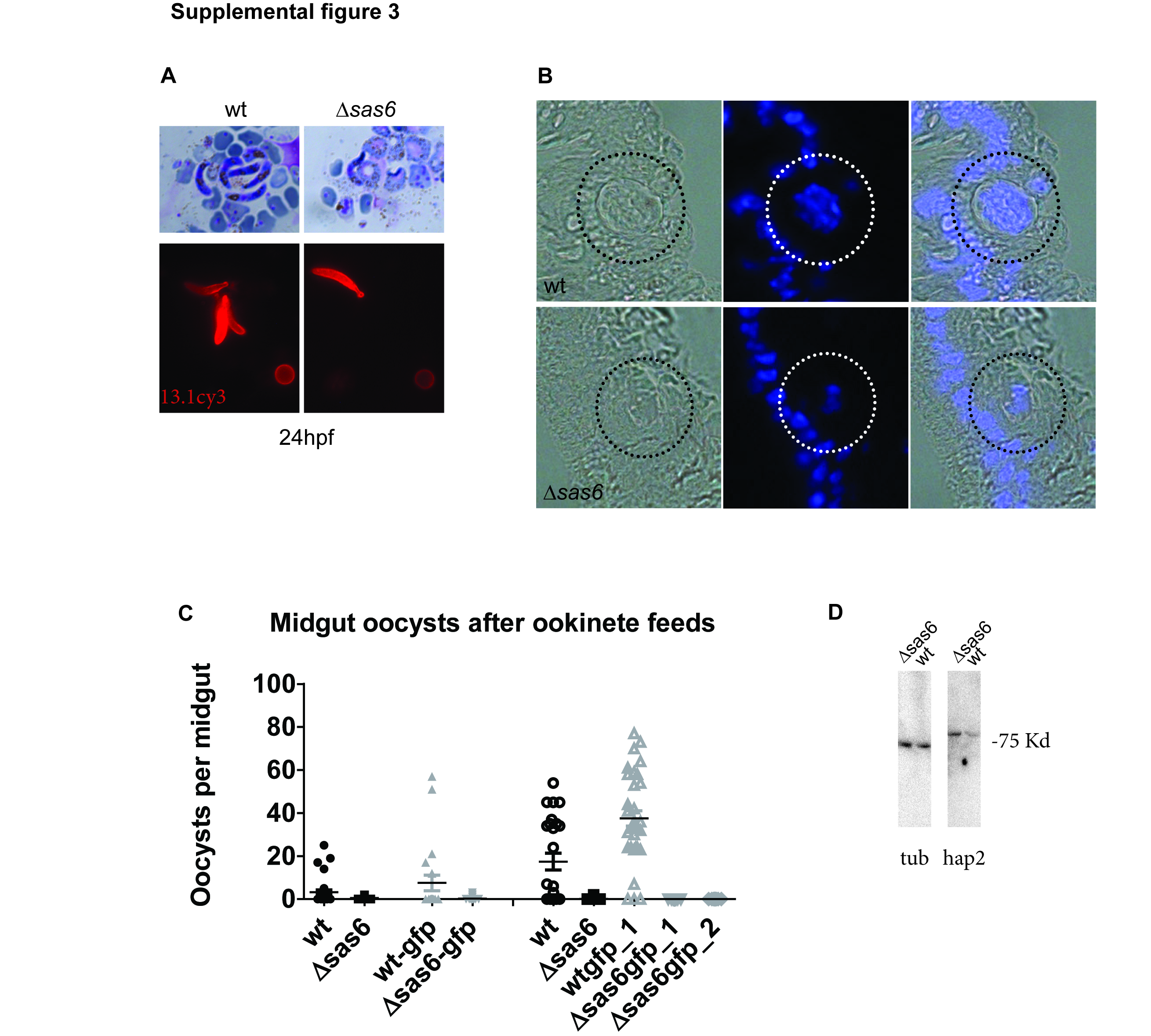

Supplement: Supplementary file 3 — Fig. S3. (A) Giemsa and P28 stainings using 13.1 labelled Cy3 of activated females and ookinetes at 24 h post fertilization (hpf). Δsas6 activated females and ookinetes display regular morphology and express P28 suggesting these parasite stages are indistinguishable from wt. (B) Oocyst images at day 12/13. Infected midguts were embedded in agar and wax, 4 micron sections were taken dehydrated and stained with DAPI. Wt oocysts display wide diameters and their DNA displays a distinct punctated arrangement, Δsas6 oocysts are smaller and DNA appears disorganized and diffuse. Dotted lines outline the oocysts (C) Ookinete fed mosquito infections, oocyst counts were performed at day 9 and 13 post-feed, prevalence and ookinete concentration is shown in Table S3 (D) Hap2 presence in purified activated gametocytes. Westernblot analysis showing that fusogenic protein Hap2 is present in Δsas6. [file cmi0017-0191-sd3.tif]
