# Supplementary material for: An essential role of the basal body protein SAS-6 in Plasmodium male gamete development and malaria transmission
Source: Cell Microbiol. 2014 Sep 24;17(2):191–206. doi: 10.1111/cmi.12355 (PMC4441282; doi:10.1111/cmi.12355)
Supplement: Supplementary file 4 [file cmi0017-0191-sd4.docx]

**Supplemental Tables**

**Supplemental Table 1. *In vitro* ookinete conversion assay**

| **Genotype** | **Conversion rate** | |
| --- | --- | --- |
| wt-gfp | 87.4±4.9 | |
| *∆sas6-gfp* | 5.5±3.1* | |
| wt | 55.1±9.2 | |
| *∆sas6* | 2.8±0.5* | |
| *∆cdpk4* | 1.1±0.3 | |
| *∆nek4* | 0 | |
| *∆nek4X∆cdpk4* | 35.3±6.6* | |
| *∆sas6X∆cdpk4* | 1.3±0.4 | |
| *∆sas6X∆nek4* | 30.9±10.3* | |
|  | |  |

For each replicate, conversion rate was calculated as the percentage of macrogametes that developed into ookinetes. Values represent average of more than 800 cells counted. Asterisk * indicate statistically significant differences in Student’s T-test with p-values lower than 0.01.

**Supplemental Table 2A.** **Intensity and prevalence of mosquito infections – day 6**

| **Genotype** | **Prevalence**  **(% infected *An.gambiae* mosquitoes)** | **Mean Intensity**  **(ookinete** **number/ midgut)** | **Range of ookinete number** | **N**  **(number of mosquitoes)** |
| --- | --- | --- | --- | --- |
| **wt** | 100  89  95 | 28.2  66.6  36.2 | 5-74  0-184  0-148 | 16  19  23 |
| ***∆sas6*** | 50*  14*  95 | 2*  0.1*  7.1* | 0-10  0-1  0-62 | 22  7  21 |

**Supplemental Table 2B. Intensity and prevalence of mosquito infections – day 12**

| **Genotype** | **Prevalence**  **(% infected *An.stephensi* mosquitoes)** | **Intensity average**  **(oocyst number/ midgut)** | **Range of oocyst number** | **N**  **(number of mosquitoes)** |
| --- | --- | --- | --- | --- |
| **wt** | 96  98  94 | 75.80  120.46  141.08 | 0-330  0-320  0-426 | 50  50  50 |
| ***∆sas6*** | 0*  3.9*  5.9* | 0*  0.06*  0.06* | 0  0-1  0-1 | 50  50  50 |
| **wt-gfp** | 98  98 | 505.7  474.3 | 0-913  0-878 | 45  44 |
| ***∆sas6-gfp*** | 3.8*  2.2* | 0.04*  0.02* | 0-2  0-1 | 51  45 |

For each replicate, mice with similar parasitaemias were fed to mosquitoes. Fully fed mosquitoes were kept until midgut dissection, midguts were mounted on slides and melanised ookinetes or oocysts were counted under the microscope. Differences between groups were calculated with Fisher’s exact test for prevalence, Mann-Whitney test for intensity. Asterisk * indicate statistically significant differences with p-values lower than 0.0001 and 0.005, respectively.

**Supplemental Table 3. Intensity and prevalence of mosquito infections from ookinete feeds**

| **Genotype/**  **Oocyst day** | **Ookinete concentration** | **Prevalence**  **(% infected *An.stephensi* mosquitoes)** | **N**  **(number of mosquitoes)** | **Oocyst size in ImageJ arbitrary units/ number of oocysts** |
| --- | --- | --- | --- | --- |
| **wt/ day 9** | 8 ook/ul | 58 | 34 | 6012 ± 1661/32 |
| ***∆sas6/day 9*** |  | 46 | 37 | 2179±1178*/22 |
| **wt-gfp/day 9** | 5 ook/ul | 23 | 22 |  |
| ***∆sas6-gfp/*day 9** |  | 16 | 23 |  |
| **wt/ day 13** | 12 ook/ul | 52 | 26 |  |
| ***∆sas6/*day 13** |  | 3.3* | 30 |  |
| **wt-gfp/day 13** | 15 ook/ul | 90 | 34 |  |
| ***∆sas6-gfp/*day 13** |  | 0* | 38 |  |
| ***∆sas6-gfp/*day 13** |  | 0* | 30 |  |

For each experiment, similar concentrations of either wt or knockout purified ookinetes were fed to mosquitoes. Fully fed mosquitoes were kept until midgut dissection, midguts were mounted on slides and oocysts were counted under the microscope. Differences between groups were calculated with Fisher’s exact test for prevalence and T-test for oocysts diameters. Asterisk * indicate statistically significant differences with p-values lower than 0.005.

**Supplemental Table 4. Cloning, diagnostic integration and RT primers**

| **Cloning constructs** | **Primer** |
| --- | --- |
| GTG GTA CCA CAC TTT TAA GGC TAC TTC TG | **A** |
| CGC GGG CCC ACA TAT GCT TTT CTT TTC TAA TTA CTG | **B** |
| GTT A GGATCC GTG CTT ATT ATA TCT GCA CTT CCC | **C** |
| CCT CTC TAGA T TTA TTG TTA CAT ATC CAC | **D** |
| GTT AAG GTA CCA GAT TTG AAC ATA TCT CGA GAG GGT | **E** |
| AAT TGG GCC CGG GAG GAA TAA ATT TCA CGG | **F** |
| GCC AAT TTA AAG ATA TCA TCA GGA ATA GGT TAC | **F- EcoRV** |
| GTA ACC TAT TCC TGA TGA TAT CTT TAA ATT GGC | **R - EcoRV** |
| **Integration constructs** |  |
| CTA TGA TTA TTT TCT TAT AAA GAA TGT C | **1** |
| GAT GTG TTA TGT GAT TAA TTC ATA CAC | **2** |
| CAA TGC ATA AAC CGG TGT GT | **3** |
| CCT AGA CAG CCA TCT CCA T | **4** |
| GTG CAT GCA CAT GCA TGT AAA TAG C | **5** |
| GGA CAA AGA ATC GGC CAT TAG CAG ATG GAT C | **6** |
| GCC AAT TTA AAG ATA TCA TCA GGA ATA GGT TAC | **7** |
| AAT TGG GCC CGG GAG GAA TAA ATT TCA CGG | **8** |
| GAT AAC CTA CGA CAA GAA GTA AAT ACT TTA C | **9** |
| TAA GTC TTC CTC ACT TAT TAA | **10** |
| **RT-PCR constructs** |  |
| GTT AAA GAT TTG AAC ATA TCT CGA GAG G | **RTsas-6F** |
| CCG GGA GGA ATA AAT TTC ACG G | **RTsas-6F** |
| CCAGATGGTCAAATGCCC | **RTαtubF** |
| CT GTGGTGATGGCCATGAAC | **RTαtubR** |
